# Acute Pancreatitis and Splenic Vein Thrombosis due to Hypertriglyceridemia

**DOI:** 10.1155/2015/729510

**Published:** 2015-02-23

**Authors:** Ercan Gündüz, Recep Dursun, Mustafa İçer, Yılmaz Zengin, Cahfer Güloğlu

**Affiliations:** Department of Emergency Medicine, Dicle University, Diyarbakır, Turkey

## Abstract

Acute pancreatitis (AP) is a condition characterised by the activation of the normally inactive digestive enzymes due to an etiological factor and digestion of the pancreatic tissues, resulting in extensive inflammation and leading to local, regional, and systemic complications in the organism. It may vary from the mild edematous to the hemorrhagic and severely necrotising form. The most common causes are biliary stones and alcohol abuse. In this case study, we would like to present a patient with AP due to hypertriglyceridemia (HPTG), which is a rare cause of pancreatitis, and splenic vein thrombosis, which is a rare complication of pancreatitis.

## 1. Introduction

Acute pancreatitis (AP) occurs due to the autodigestion of the pancreas due to the activation of the digestive enzymes within the pancreas. It is an acute inflammatory process characterised by severe pain localised in the upper quadrant of the abdomen which may be radiating towards the back and the condition may be accompanied by systemic findings primarily including the surrounding organs [1]. While alcohol consumption and biliary pathologies are among the most common causes of AP, AP due to hypertriglyceridemia (HPTG) is still considered to be rare in spite of the frequencies reported between 12% and 38% [2]. The probable mechanism of AP in patients with HPTG is the excessive release of local free fatty acids and lysolecithin from the lipoprotein substrates in the pancreatic bed, leading to an overload of the loading capacity of the albumin and to damage in the acinar cells and microvascular membranes [3]. HPTG is considered a risk for pancreatitis when levels are >1000 mg/dL (11.2 mmol/L) [4]. The literature information about the treatment and follow-up of AP that develops on this background is limited. In this report, our aim is to present a case of AP that developed due to HPTG and led to the complication of splenic vein thrombosis in the light of the current information.

## 2. Case Presentation

**Figure 1:**
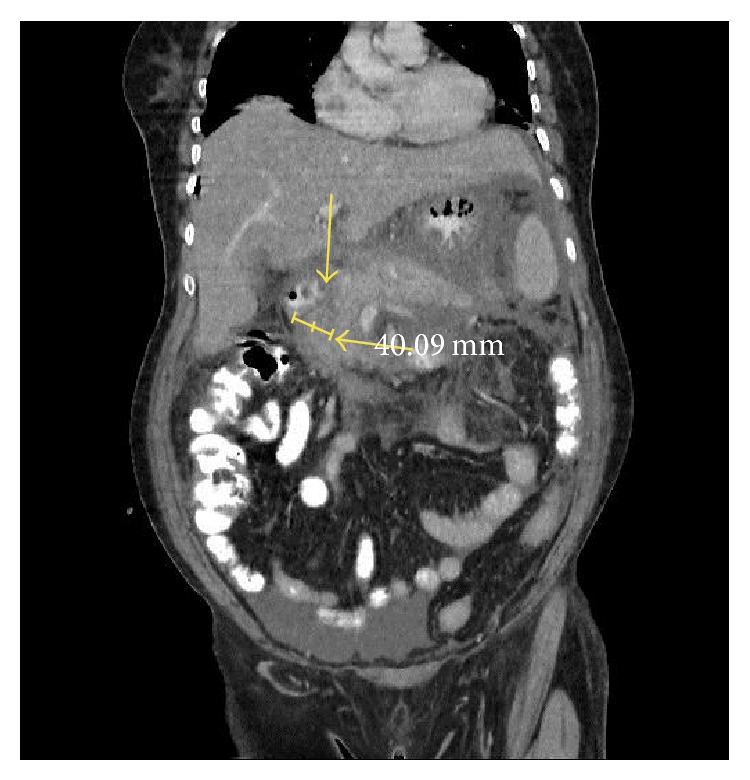
Arrows point to the diffuse enlargement in the size of the pancreas (40.09 mm) (AP) (normal size of pancreas 10 mm up to 30 mm).

**Figure 2:**
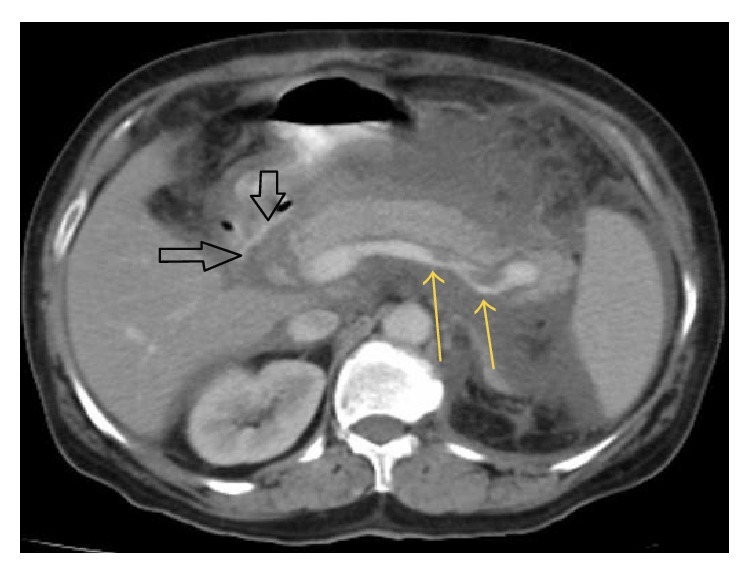
The smaller yellow arrows indicate the filing defect (thrombosis) in the splenic vein and large black arrows point to necrotic areas of head of pancreas.

A 65-year-old female patient presented to our emergency room with abdominal pain, nausea, vomiting, and pain radiating towards the back. The patient history included nothing specific other than diabetes mellitus (DM) for 20 years and cholecystectomy a year ago. She did not have a history of alcohol consumption and she was on subcutaneous insulin treatment twice a day. During the physical examination, the patient was observed to be in a confused state and her general state was poor; her blood pressure was 90/55 mmHg, heart rate was 132/min, body temperature was 38.3°C, and the respiratory rate was 28/min. Her abdomen was slightly distended and epigastric tenderness was detected. No rebound or guarding was observed. The laboratory parameters were as follows: Hb: 17.1 g/dL, Hct: 45.7%, WBC: 8.9 K/uL, PLT: 505 K/uL, sedimentation: 37/hour, CRP: 24 mg/dL, glucose: 255 mg/dL, urea: 28 mg/dL, creatinine: 1.6 mg/dL, AST: 57 U/L, ALT: 34 U/L, LDH: 1995 U/L, amylase: 212 U/L, lipase: 349 U/L, triglycerides: 2148 mg/dL, albumin: 2.3 g/dL, calcium: 6.5 mg/dL, and HbA1c: 9.6%. The blood gas analyses gave the following results: pH: 7.32 mm/Hg, pCO2: 44 mm/Hg, PO2: 72 mm/Hg, HCO3: 19.6 mmol/L, and lactate: 0.9 mmol/L. The other laboratory parameters were normal. The abdominal ultrasonography was interpreted in favour of AP. In the abdominopelvic contrast-enhanced computed tomography (CT), the gall bladder could not be observed (excised) and the pancreas was diffusely enlarged (Figure 1), while widespread increases of density and fluid retention were observed in the peripancreatic bilateral anterior renal fascia, splenic hilum, mesenteric adipose tissue, and the pelvic region. There was 19 × 18 mm necrosis area in the head of pancreas. Filling defects (thromboses) were observed in the splenic vein at the level of the tail of the pancreas. The patient's CT image shows the AP and splenic vein thrombosis (Figure 2). A central venous catheter was placed and the patient was prescribed blood glucose tests every six hours and insulin treatment for the regulation of her blood glucose levels. After the consultation with the infectious diseases department, the patient was started on 4 × 500 mg/day of imipenem. She was administered 4 × 5000 units of low molecular weight heparin through the subcutaneous route and infused intravenously 5 units of regular insulin along with each 1000 mL 5% dextrose fluid and lipid apheresis was performed once a day for three days. After the three times lipid apheresis, the triglyceride values declined to 829 mg/dL. In addition to the low molecular weight heparin, the patient was started on warfarin treatment and the dose was adjusted to keep the patient's INR value over 2. When the patient's general condition was observed to improve after the treatment, she was discharged with the recommendation of a low-fat diet and fenofibrate and warfarin treatment.

## 3. Discussion

Although different studies have reported different prevalence ratios for HPTG in AP, the frequency is similar in edematous and necrotizing pancreatitis [5]. HPTG may be both an etiological factor and a result of AP [6]. Triglyceride levels of 1000 mg/dL or above may trigger acute pancreatitis and the serum of these patients has the consistency of milk due to the increased LDL-cholesterol [4]. AP due to HPTG occurs in three groups of patients comprising diabetic patients with poor glycemic control, alcoholic patients, and those who have HPTG due to diet or drugs [7]. Type 2 DM causes moderate HPTG and the total cholesterol and LDL cholesterol levels may be higher. The level of HPTG in these patients is parallel to the depravity of insulin which is an LPL activator [8]. In our patient, the HbA1c value was 9.6 (4.5–6.6) and the etiology of HPTG was associated with the poorly regulated blood glucose levels in type 2 DM. Since the serum amylase levels in hyperlipidemic plasma (hypertriglyceridemia) may give lower results, high serum amylase values may not be observed during the pancreatitis attack in these patients [9]. The complications of AP include sterile pancreatic necrosis, infected pancreatic necrosis, chronic pancreatitis, pancreatic pseudocysts, pancreatic fistulas, splenic vein thrombosis, pancreatic ascites, and pseudoaneurysm in the splenic artery or gastroduodenal artery and renal failure, hypocalcemia, diffuse fat necrosis, and adult respiratory distress syndrome [10]. Coagulation disorders in AP vary from mild intravenous thrombosis to disseminated intravenous coagulation. Twenty years ago Shinovvara suggested that hypercoagulability in AP is associated with higher plasma levels of fibrinogen and factor VIII [11]. In our patient, splenic vein thrombosis was detected and it is a rare complication of AP. Considering that our patient has had DM for 20 years, the microvascular complications associated with DM may be thought to increase the tendency of thrombosis. Since our patient was 65 years old, hereditary thrombophilia was not considered as an etiological factor of the thrombosis. In a previous study, the frequency of splenic vein thrombosis in pancreatitis due to HPTG was reported as 3.6% [12]. The treatment of pancreatitis due to HPTG is controversial. In the literature, insulin that exerts its activity by increasing the lipoprotein lipase activity and/or heparin as well as apheresis to remove the triglycerides are the most frequently applied treatment alternatives [13]. In our patient, low molecular weight heparin was coadministered with warfarin and apheresis treatment. After the apheresis, the patient's triglyceride level was significantly reduced and her laboratory values and clinical status were improved. Due to the splenic thrombosis, the patient was treated with warfarin maintaining INR levels over 2 and was referred for outpatient care.

In conclusion, in patients with AP who are diabetic or obese or consume alcohol, HPTG should be considered as the etiology and splenic thrombosis as a complication. In addition to the usual measures, apheresis is an efficient therapeutic method.
